# Subclinical Atherosclerosis Measure by Carotid Ultrasound and Inflammatory Activity in Patients with Rheumatoid Arthritis and Spondylarthritis

**DOI:** 10.3390/jcm11030662

**Published:** 2022-01-27

**Authors:** Marta Rojas-Giménez, Clementina López-Medina, María Lourdes Ladehesa-Pineda, María Ángeles Puche-Larrubia, Ignacio Gómez-García, Jerusalem Calvo-Gutiérrez, Pedro Seguí-Azpilcueta, María del Carmen Ábalos-Aguilera, Desirée Ruíz-Vilchez, Alejandro Escudero-Contreras, Eduardo Collantes-Estévez

**Affiliations:** 1 Rheumatology Department, Reina Sofia University Hospital, Maimonides Biomedical Research Institute of Cordoba (IMIBIC), University of Córdoba (UCO), 14004 Cordoba, Spain; rojasgimenezm@gmail.com (M.R.-G.); lourdesladehesapineda@gmail.com (M.L.L.-P.); mangeles.puche@gmail.com (M.Á.P.-L.); ignaciogomgar@gmail.com (I.G.-G.); yeru83@hotmail.com (J.C.-G.); mc.abalos@outlook.com (M.d.C.Á.-A.); desiree.ruiz@imibic.org (D.R.-V.); alexcudero2@gmail.com (A.E.-C.); educollantes@yahoo.es (E.C.-E.); 2 Radiology Department, Reina Sofia University Hospital, Maimonides Biomedical Research Institute of Cordoba (IMIBIC), 14004 Cordoba, Spain; pedromsegui@telefonica.net

**Keywords:** rheumatoid arthritis, spondyloarthritis, subclinical atherosclerosis, cardiovascular risk, inflammatory activity

## Abstract

Objective: To compare the effect of inflammation on subclinical atherosclerosis using carotid ultrasound in patients with rheumatoid arthritis (RA) and spondyloarthritis (SpA). Methods: Cross-sectional study including 347 participants (148 RA, 159 SpA, and 40 controls). We measured the carotid intima media thickness (cIMT) and detection of atheromatous plaques using carotid ultrasound. We recorded disease activity (DAS28-CRP/ASDAS-CRP) and traditional cardiovascular risk factors. We performed descriptive, bivariate, and linear multivariate analyses (dependent variable: cIMT) to evaluate the influence of diagnosis on cIMT in all patients. Two additional multivariate analyses were performed by stratifying patients according to their inflammatory activity. Results: cIMT correlated with the mean CRP during the previous 5 years in RA, but not with CRP at the cut-off date. We did not find such differences in patients with SpA. The first multivariate model revealed that increased cIMT was more common in patients with RA than in those with SpA (β coefficient, 0.045; 95% confidence interval (95% CI), 0.0002–0.09; *p* = 0.048) after adjusting for age, sex, disease course, and differential cardiovascular risk factors (arterial hypertension, smoking, statins, and corticosteroids). The second model revealed no differences in cIMT between the 2 groups of patients classified as remission–low activity (β coefficient, 0.020; 95% CI, −0.03 to 0.080; *p* = 0.500). However, when only patients with moderate–high disease activity were analysed, the cIMT was 0.112 mm greater in those with RA (95% CI, 0.013–0.212; *p* = 0.026) than in those with SpA after adjusting for the same variables. Conclusions: Subclinical atherosclerosis measured by carotid ultrasound in patients with RA and SpA is comparable when the disease is well controlled. However, when patients have moderate–high disease activity, cIMT is greater in patients with RA than in those with SpA after adjusting for age, sex, disease course, and cardiovascular risk factors. Our results point to greater involvement of disease activity in subclinical atherosclerosis in patients with RA than in those with SpA.

## 1. Introduction

Patients with rheumatoid arthritis (RA) have high cardiovascular morbidity and mortality, with a relative risk of a cardiovascular episode that is approximately double that of a healthy individual of the same age and sex [1]. These conditions include ischaemic heart disease and cerebrovascular accidents [2]. Spondyloarthritis (SpA) comprises a series of chronic inflammatory diseases whose most frequent comorbidities are characterized mainly by osteoporosis [3] but also by cardiovascular conditions, especially atherosclerosis. Affected patients have a greater risk of cardiovascular events than the general population [4]. This increased risk in both diseases is due to a process of accelerated atherogenesis [5] that can be affected by both traditional and non-traditional cardiovascular risk factors (CVRFs), including systemic inflammation and dyslipidaemia [6]. In this regard, various studies have highlighted the role of inflammation in the process of atherosclerosis, where the increased expression of proinflammatory cytokines and adhesion molecules (e.g., tumour necrosis factor alpha (TNF-α) and interleukin 6 (IL-6)) [7] leads to endothelial dysfunction, promotes the formation of atheromatous plaques, and increases the vulnerability to this condition. Therefore, inflammation acts as a key player in each phase of atherosclerosis, which is not the only consequence of the accumulation of lipids on the arterial wall [8,9]. Given the relationship between the inflammatory response and the process of atherosclerosis, appropriate control of inflammatory activity in rheumatic diseases should be a major objective in management [10]. Therefore, a notable improvement in carotid intima media thickness (cIMT), which is used as a marker of atherosclerosis, has been reported in patients with RA once the disease is controlled [11]. Furthermore, in patients with SpA, high C-reactive protein (CRP) values at diagnosis and during the course of the disease are associated with an increased risk of cardiovascular events and mortality from such events. Therefore, better control of disease and inflammation is expected to reduce this risk [12].

Various studies have suggested that cardiovascular risk is underestimated when calculated using scales or scores recommended by guidelines [13]; therefore, complementary techniques could help to improve the estimation of this risk. Ultrasound assessment of cIMT and atheromatous plaques has been reported to be a useful tool for detecting subclinical atherosclerosis in patients with RA or SpA [14,15]. Even in the absence of atheromatous plaques, increased cIMT has been determined as a predictor of CV events, as well as a marker of subclinical atherosclerosis, and has been used in clinical trials as an alternative assessment method or a surrogate endpoint and can predict the appearance of plaques with significant accuracy [16,17].

Clearly, both diseases carry an increased cardiovascular risk, and the inflammatory process plays a key role. A study from the CARMA Project (CARdiovascular in rheuMAtology Project) revealed an odds ratio for cardiovascular disease (vs. healthy controls) of 1.77 (95% CI, 0.96–3.27; *p* = 0.07) in patients with SpA and 1.58 (95% CI, 0.90–2.76; *p* = 0.10) in patients with RA [18]. However, we do not know whether the frequency of this inflammatory process and of subclinical atherosclerosis is equally increased in both diseases, since no studies have compared them. Therefore, the objectives of this study were to evaluate and compare the cardiovascular risk associated with both diseases using carotid ultrasound and to analyse the effect of disease activity on cIMT.

## 2. Patients and Methods

### 2.1. Design

We performed a cross-sectional observational study of a cohort of patients with RA from our unit and a cohort of patients with SpA from the Córdoba Spondyloarthritis Task Force, Registry and Outcomes (CASTRO), which was performed in the Department of Rheumatology of at the Reina Sofía University Hospital and Maimonides Institute of Biomedical Research of Cordoba (IMIBIC). All participants signed informed consent before being included in the study.

### 2.2. Study Population and Protocol

We consecutively enrolled patients aged >16 years diagnosed with RA according to the criteria of American College of Rheumatology/European League Against Rheumatism 2010 [19] or with a diagnosis of SpA according to the criteria of the Assessment of Spondylarthritis International Society [20]. We excluded patients with other, concomitant rheumatic inflammatory diseases or active infection and patients who were pregnant. For the control group without rheumatic inflammatory disease, participants were selected consecutively from the persons accompanying the patients. Fasting blood samples were taken from all participants who also underwent physical examination (according to a pre-established protocol) and carotid ultrasound. The study was approved by the Clinical Research Ethics Committee of Hospital Universitario Reina Sofía (date, 29 July 2021; code no., 1820-N-21).

### 2.3. Variables

The main variable was cIMT assessed with B-mode ultrasound performed by a unique expert radiologist using a Philips Epiq-7 system with a broadband 5–14 MHz transducer. Ultrasound enabled measurement of cIMT and detection of atheromatous plaques, which were defined by consensus [21,22] as focal thickening on the arterial wall protruding towards the lumen and measuring >0.5 mm or more than 50% of the neighbouring cIMT, or when the cIMT was >1.5 mm.

A series of laboratory variables were collected: total cholesterol, triglycerides, LDL and HDL cholesterol, homocysteine (normal, 0.68–1.62 mg/L), glucose, apolipoprotein B (normal, 65–130 mg/dL) and A1 (normal, 105–220 mg/dL), apoB/apoA1 ratio, rheumatoid factor (RF) (positive if >14 IU/mL), anticyclic citrullinated peptide antibody (ACPA) (positive if >7 IU/mL), and HLA-B27. The erythrocyte sedimentation rate (ESR) (normal, <20 mm/h) and CRP (normal, <10 mg/dL) were evaluated both at the study visit and retrospectively for the previous 5 years (cumulative CRP). Mean CRP values were calculated for all the tests performed in the hospital during the previous 5 years. We recorded retrospectively once, twice, or three times per year during the 5 years prior to study and at the moment of the study, so at least six determinations of CRP levels for each patient were available if the disease duration was greater than 5 years. Patients were considered to have persistent inflammation when they had high CRP levels in at least 50% of determinations during the previous 5 years.

Data on inflammatory activity were collected. The 28-joint Disease Activity Score with CRP (DAS28-CRP), Simplified Disease Activity Index (SDAI), and Clinical Disease Activity Index (CDAI) were calculated at the study visit for patients with RA, and the Ankylosing Spondylitis Disease Activity Score (ASDAS-CRP) was calculated for patients with SpA. Patients were classified as remission–low activity if the DAS28 was ≤3.2 or the ASDAS was <2.1 and moderate–high activity if the DAS28 was >3.2 or the ASDAS was ≥2.1. Severity scores such as the presence of radiological erosions or sacroiliitis on an X-ray were evaluated by a trained local reader.

Sex and the following traditional CVRFs were also recorded: smoking, body mass index (BMI) (kg/m^2^), arterial hypertension, diabetes mellitus diagnosed according to the recommendations of the American Diabetes Association [23], and a personal history of cardiovascular disease (ischaemic heart disease or cerebrovascular accident). Finally, data were collected on treatment administered up to the date of the study visit: conventional synthetic disease-modifying antirheumatic drugs (csDMARDs) and/or biologic DMARDs (bDMARDs), statins, and corticosteroids.

### 2.4. Statistical Analysis

First, we performed a descriptive analysis. Qualitative variables are expressed as absolute numbers and percentages; quantitative variables are expressed as the mean and standard deviation (SD). An χ^2^ test was performed to compare categorical variables, a *t*-test for independent samples to compare mean values of the continuous variables, and an analysis of variance (ANOVA) for comparisons of mean values in more than two groups. We compared baseline variables, comorbid conditions, and laboratory data between RA and controls, SpA and controls, and RA and SpA groups. Linear correlation analyses (Pearson correlation coefficients) were then performed to evaluate the association between cIMT and various clinical variables, both in patients with RA and patients with SpA.

Multivariate linear regression models were run (dependent variable: cIMT) to evaluate the influence of diagnosis on cIMT in the whole group of patients adjusted for age, sex, disease course, and CVRFs.

We ran the same multivariate linear regression models, with patients stratified by inflammatory activity (remission–low activity if DAS28 ≤ 3.2 or ASDAS < 2.1 and moderate–high activity if DAS28 > 3.2 or ASDAS ≥ 2.1) to determine whether the association between diagnosis and cIMT depended on the disease activity. Finally, we constructed logistic regression models with the presence of carotid atheromatous plaques as the dependent variable.

The 95% confidence interval (CI) was estimated, and statistical significance was set at *p* ≤ 0.05 for all the analyses. The analyses were performed using R Studio 1.4.1106, RStudio, Boston, MA, US.

## 3. Results

### 3.1. Demographic, Laboratory, and Disease-Related Data

The study population comprised 347 participants (148 RA, 159 SpA (87% axial SpA and 13% peripheral SpA), and 40 controls). Table 1 shows the baseline and clinical characteristics and comorbidities for each group. The mean age of RA patients was higher than that of the other groups, as was the prevalence of hypertension and dyslipidaemia. Patients with SpA were more frequently smokers and had a longer disease history.

While patients with RA had higher total, apoB, and LDL cholesterol levels, the atherogenic ratio was no higher than that in patients with SpA, since they also had higher levels of apoA1 and HDL cholesterol.

For treatment, 51.3% of patients with RA were receiving biologics (mainly anti-TNFα, agents (18.9%) and anti-IL6 agents (14.8%)) compared with 1.9% of patients with SpA (*p* < 0.001). Patients with RA were also more frequently treated with statins and corticosteroids than patients with SpA (*p* = 0.002 and *p* < 0.001, respectively).

Regarding inflammatory activity at the study visit, 56.7% of patients with RA were classified as remission–low activity compared with 37.1% of patients with SpA (*p* < 0.001). However, 22.7% of patients with RA had persistent inflammation as measured by CRP, compared with 11.3% of those with SpA (*p* = 0.008) during the 5 years prior to the cut-off date, as well as higher CRP levels at the cut-off (*p* = 0.029) and a greater mean CRP value during the previous 5 years (*p* < 0.001).

### 3.2. Association between Cardiovascular Risk and Laboratory Markers

Patients with RA had a higher mean cIMT than the other two groups (*p* < 0.001). In general, atheromatous plaques were more frequent in patients with RA and patients with SpA than in the control group (*p* < 0.001); the frequency was higher for those with RA than for those with SpA (*p* = 0.006). Patients with radiographic axial SpA (r-axSpA) had similar cIMT to non-radiographic (mean (SD); 0.56 mm (0.13) vs. 0.53 mm (0.10), respectively; *p* = 0.245) but more patients with r-axSpA had atheromatous plaques (*n* (%); 29 (24.4) vs. 4 (10%), respectively; *p* = 0.050). Patients with RA continued to have higher cIMT than patients with r-axSpA (*p* < 0.001) but with no differences found in the atheromatous plaques (*p* = 0.075).

When the correlation analyses were performed on the entire samples, cIMT was associated with the mean CRP value during the previous 5 years (r = 0.181; *p* = 0.002), although we did not observe this correlation with CRP at the cut-off date (r = 0.180; *p* = 0.062). In patients with RA, cIMT was also associated with the mean CRP during the previous 5 years, although not with CRP at the study visit or with disease activity according to DAS28. Similarly, we did not observe a correlation with RF (r = 0.08; *p* = 0.321) or ACPA (r = 0.135; *p* = 0.111). cIMT was comparable between patients with erosions and those without erosions (mean (SD) 0.65 mm (0.23) vs. 0.63 mm (0.15), respectively; *p* = 0.559).

However, in patients with SpA, statistical analyses did not reveal an association between cIMT and the mean CRP (r = 0.04; *p* = 0.626), CRP at the cut-off date (r = −0.116; *p* = 0.149), or ASDAS-CRP (r = 0.06; *p* = 0.467). Similarly, no differences were found in cIMT between patients positive and negative for HLA-B27 (mean (SD), 0.56 mm (0.12) vs. 0.53 mm (0.14), respectively; *p* = 0.316).

The multivariate analysis adjusted for sex, age, disease course, and traditional CVRFs confirmed that cIMT was higher in patients with RA than in those with SpA (β coefficient, 0.045; 95% CI, 0.0004–0.09; *p* = 0.048) (Table 2).

### 3.3. Cardiovascular Risk According to Inflammatory Activity

Next, we stratified patients according to inflammatory activity (DAS28 or ASDAS). Among patients with moderate–high activity (DAS28 > 3.2 or ASDAS ≥ 2.1), cIMT was greater in those with RA than in those with SpA (mean (SD), 0.65 mm (0.20) vs. 0.55 mm (0.13), respectively; *p* = 0.003). This same result was obtained with patients classified as remission–low activity (DAS28 ≤ 3.2 or ASDAS < 2.1) (mean (SD), 0.63 mm (0.18) vs. 0.54 mm (0.11) in patients with RA and SpA, respectively; *p* < 0.001).

However, when the results were adjusted for age, sex, disease course, and traditional CVRFs, we found no differences in cIMT between the diseases when patients were classified as remission–low activity (RA vs. SpA, β coefficient, 0.020; 95% CI, −0.039 to 0.080; *p* = 0.442) (Table 3). When only patients with moderate–high activity were analysed (Table 4), the cIMT was 0.11 mm greater in those with RA (95% CI, 0.013–0.21; *p* = 0.026) than in those with SpA, irrespective of the previous variables.

The same analyses were performed with the presence of atheromatous plaques as the dependent variable. However, the models adjusted for the previous variables did not reveal differences in the presence of atheromatous plaques between the diseases according to inflammatory activity (Supplementary Materials Table S1).

## 4. Discussion

Various studies have shown that patients with RA or SpA have a higher cardiovascular risk—measured using carotid ultrasound—than the general population [24]. However, to our knowledge, no studies have compared these two diseases. Our results show that in patients with moderate–high disease activity, cIMT is greater in patients with RA than in those with SpA, irrespective of age, sex, disease course, and traditional CVRFs. This could lead to an increased likelihood of future cardiovascular events.

Very few studies have compared the prevalence of traditional CVRFs between rheumatic diseases. A recent study from Sweden [25] found that hypertension and dyslipidaemia were more common in patients with RA than in those with SpA. In our setting, the CARMA study, which was performed in Spanish patients, reported similar results and found that smoking was more prevalent in patients with SpA than in those with RA [18]. Our findings support these results, although with no differences in the prevalence of diabetes mellitus or the presence of previous cardiovascular events.

With respect to lipid values, we found higher levels of total, apoB, and LDL cholesterol in patients with RA than in those with SpA, although we also observed an increase in apoA1 and HDL. Our results are consistent with previous reports that, in patients with RA, there is a paradoxical association between lipids and disease and that control of inflammation can increase lipid values in serum without affecting atherogenic lipid ratios [26]. Our results support this observation; more than half of the patients with RA were classified as remission–low activity.

A recent study found an association between structural damage—measured using mSASSS—and cardiovascular risk in patients with SpA [27]. We did not identify an association between sacroiliitis-related damage on X-ray and cIMT, but we found that there were more patients with atheromatous plaques in the group with r-axSpA than in the group of patients without radiographic involvement. Patients with non-radiographic axial SpA present a weaker inflammatory response; however, it is not clear whether they have a higher CV risk similar to that reported in r-axSpA. A recently published multicentre study of 806 patients found no differences in the prevalence of carotid plaques or in the cIMT between both groups in the adjusted analysis [28].

The frequency of RF and ACPA, as well as the presence of radiographic erosions in patients with RA, were similar to findings reported from other series of patients with RA in Spain [29]. However, no significant association was found between these data and cIMT, contrary to prior observations in smaller cohorts of patients with RA [30]. The frequency of HLA-B27 was also comparable to that reported elsewhere [31], with no association identified between this parameter and cIMT.

Patients with RA in the present study generally had higher levels of inflammatory markers and greater cIMT than patients with SpA, even though most were receiving biologic therapy, specifically anti-TNFα agents. While there is no full consensus in this respect, various studies suggest that treatment with anti-TNFα agents could reduce cIMT and, in turn, cardiovascular risk [32,33]. In our study, we did not include the intake of non-steroidal anti-inflammatory drugs (NSAIDs). SpA patients usually take these drugs chronically, but RA patients use these medications on demand, so controlling their use is very difficult, and a count is not always reliable. Some studies evaluate the effect of different treatments on cIMT; Kim et al. found no relationship between cIMT in RA patients and taking NSAIDs [34]. In SpA, the impact of anti-inflammatory drugs on CV events or mortality has rarely been assessed [35].

CRP, a marker of acute inflammation, has been considered a marker for atherosclerosis and has proven useful for predicting cardiovascular disease in the general population [36], as well as in patients with rheumatic disease. Some studies have found a linear correlation between CRP values and cIMT in patients with RA [37,38]. While the association between cardiovascular risk and inflammatory burden is well documented in RA [39,40], few studies have evaluated this association in SpA. In a previous study [27], we found that patients with SpA and persistent inflammation as defined by CRP had greater right and left cIMT, thus pointing to a link between CRP and subclinical atherosclerosis. Notably, other studies used a single measurement of CRP at the time of the study for their analyses. In contrast, we included the mean CRP of all measurements during the previous 5 years. We believe this can provide more information than an isolated measurement, since cardiovascular risk does not result from a single factor, in this case inflammation, at a specific time point, but from a cumulative inflammatory process, together with other risk factors [41,42]. In fact, we found a linear association between the mean CRP during the previous 5 years and cIMT, although no such association was found between a single CRP value and cIMT at the cut-off date.

While the inflammatory burden defined by CRP in the present study was greater in patients with RA than in those with SpA, a greater percentage of patients with RA were classified as remission–low disease activity. However, disease activity should not be measured based on a single variable [43]. DAS28-CRP and ASDAS-CRP are the most widely used validated composite indices for evaluating the activity of these diseases in clinical practice, despite the subjective nature of some of their components [44,45]. Isolated use of the only objective component of the scores, i.e., CRP, would be inappropriate, since various studies have shown that a significant percentage of patients with normal CRP values have active disease according to overall laboratory data and clinical evaluations [46,47,48]. Therefore, we also used composite indices to classify disease activity of the patients and evaluated their association with cIMT instead of using CRP alone.

When we classified patients by activity according to these indices, we found that both groups had the same cardiovascular risk in terms of cIMT when their disease was well controlled. However, when their disease was active, cardiovascular risk was greater in patients with RA than in those with SpA. To our knowledge, this is the first study to compare cardiovascular risk in both diseases using carotid ultrasound. Our results indicate that although disease control is key to reducing cardiovascular risk in both diseases, the degree of activity has a greater effect in patients with RA than in those with SpA, irrespective of classic CVRFs, age, sex, and disease course. Therefore, our first and main objective when designing a primary cardiovascular prevention strategy should be to achieve remission–low disease activity.

While these results were obtained in relation to cIMT, when we performed the same analyses using the presence of atheromatous plaques as a reference, we did not find differences between the diseases according to inflammatory activity. This is because plaque formation constitutes a more advanced stage during the process of atherosclerosis than an increase in cIMT. The increase may also be the consequence of subclinical vasculitis and/or wall hypertrophy, as demonstrated in patients with RA [49]. cIMT without plaques has also been shown to be a key marker of an increased risk of cardiovascular events compared with the appearance of plaques [50].

One of the main limitations of our study is its cross-sectional design. At first, a cumulative measurement of the disease activity using the indices the previous 5 years might have given us more information than a single measurement with regard to association between association between cIMT and disease activity, as we have done by measuring the accumulated CRP. Furthermore, this design prevented us from evaluating whether appropriate control of inflammatory disease prevents the formation of atherosclerosis in the long term and, therefore, cardiovascular events, and whether the cardiovascular benefit of inflammation control applies to both diseases. These results can be obtained through longitudinal studies with a prospective follow-up. Various studies on either of the diseases support our hypothesis. Arida et al. performed a study with a mean follow-up of 3.2 years in which they compared 139 patients with RA in remission or with low disease activity with controls and concluded that good control of inflammatory disease could reverse any harmful effect of RA on atherosclerosis, independent of the antirheumatic or lipid-lowering treatment. Increases in cIMT during follow-up were comparable in patients with RA and controls [51]. Similar results were obtained in a meta-analysis of patients with ankylosing spondylitis [52]. Another limitation may be represented by the inclusion of patients with a previous history of a cardiovascular event, which might influence the results. However, only five patients in the RA group had a previous history of a cardiovascular event, without significant difference with the SpA group. Finally, another limitation is not having data on kidney function in our patients. Giollo et al. performed a prospective analysis evaluating the progression of subclinical atherosclerosis in SpA, and they concluded that the glomerular filtration rate and presence of syndesmophytes were associated with an accelerated atherosclerosis, independent of traditional cardiovascular risk factors [53].

Patients with RA in the present study had more traditional CVRFs than those with SpA. They were generally older and more frequently suffered from hypertension and dyslipidaemia. They were also more frequently treated with statins and corticosteroids. However, one of the strengths of the present study is that the analyses were adjusted for the CVRFs that differed between the groups. In addition, the study enrolled a large number of patients.

In conclusion, our results indicate that disease activity (measure by composite index) may play a greater role in subclinical atherosclerosis in patients with RA than in those with SpA, irrespective of age, sex, disease course, and traditional CVRFs. However, more prospective studies comparing both diseases are necessary to better determine this relationship.

## Figures and Tables

**Table 1:** Baseline clinical characteristics and comorbid conditions.

Variable	Rheumatoid Arthritis (*n* = 148) Mean (SD)	Spondyloarthritis (*n* = 159) Mean (SD)	Controls (*n* = 40) Mean (SD)	*p*-Value RA vs. Controls	*p*-Value SpA vs. Controls	*p*-Value RA vs. SpA
Baseline characteristics						
Age in years	55.8 (13.1)	45.5 (12.8)	46.87 (12)	<0.001	0.538	<0.001
Sex (female), *n* (%)	113 (76.3)	56 (35.2)	26 (65)	0.146	<0.001	<0.001
Smoking				0.218	<0.001	<0.001
Ex-smoker, *n* (%)	22 (14.8)	6 (3.8)	6 (15)			
Active smoker, *n* (%)	30 (20.3)	52 (21.6)	3 (7.5)			
BMI	27.4 (5.6)	26.6 (4.6)	24.5 (4.02)	0.006	0.035	0.174
Comorbidities						
Arterial hypertension, *n* (%)	45 (30.4)	30 (19.1)	4 (10)	0.011	0.191	0.018
Diabetes mellitus, *n* (%)	5 (3.8)	1 (0.6)	1 (2.5)	0.897	0.229	0.08
Dyslipidaemia, *n* (%)	46 (31.1)	30 (19.1)	3 (7.5)	<0.001	0.02	0.015
Previous CV events, *n* (%)	5 (3.4)	0	1 (2.5)	0.277	0.03	0.196
Disease characteristics						
Disease course (years)	8.8 (8.2)	18.9 (13.6)	-	-	-	<0.001
Erosions, *n* (%)	53 (36.6)	-	-	-	-	-
Rheumatoid factor - positive, *n* (%)	119 (81.5)	-	0	<0.001	-	-
ACPA-positive, *n* (%)	123 (84.2)	-	0	<0.001	-	-
ESR (mm/h) at cut-off	17.2 (14.7)	12.1 (17.1)	11.03 (7.5)	0.001	0.607	0.007
DAS28-CRP at cut-off	2.8 (1.2)	-	-	-	-	-
CRP (mg/dL) at cut-off	10.3 (18.6)	6.4 (10.8)	1.96 (1.9)	<0.001	<0.001	0.029
Mean CRP 5 years	10 (11.8)	5.8 (6.6)	-	-	-	<0.001
Persistently high CRP, *n* (%)	32 (22.7)	17 (11.3)	-	-	-	0.008
SDAI at cut-off	11.8 (9.3)	-	-	-	-	-
CDAI at cut-off	11.2 (8.9)	-	-	-	-	-
HLAB27-positive, *n* (%)	-	128 (82.6)	0	-	<0.001	-
Axial SpA, *n* (%)	-	136 (87.2)	-	-	-	-
Radiographic sacroiliitis, *n* (%)	-	119 (74.8)	-	-	-	-
BASDAI	-	3.8 (2.2)	-	-	-	-
BASFI	-	3.5 (2.6)	-	-	-	-
ASDAS-CRP	-	2.4 (0.9)	-	-	-	-
VAS overall	41.2 (24.5)	44.2 (26.1)	-	-	-	0.307
Remission–low activity, *n* (%)	94 (63.5)	59 (37.1)	-	-	-	<0.001
Laboratory parameters						
Glucose (mg/dL)	85.6 (13.4)	84 (12.8)	86.56 (8.7)	0.621	0.19	0.354
Total cholesterol (mg/dL)	198 (37.3)	189 (34)	202 (27.4)	0.468	0.03	0.035
LDL cholesterol (mg/dL)	118.7 (30.5)	115 (30.3)	122.3 (23.9)	0.453	0.163	0.298
HDL cholesterol (mg/dL)	59.4 (17.9)	54.5 (14.6)	58.8 (16.3)	0.881	0.152	<0.001
Cholesterol/HDL	3.5 (1.0)	3.6 (1.05)	3.4 (0.9)	0.455	0.825	0.137
Triglycerides (mg/dL)	103.8 (45.6)	101.2 (60.2)	100.8 (43.2)	0.778	0.975	0.676
Apolipoprotein A1 (mg/dL)	149.8 (32.7)	140.7 (23.2)	154.7 (26.8)	0.354	0.007	0.009
Apolipoprotein B (mg/dL)	86.8 (21.8)	80.4 (17.7)	84.5 (15.5)	0.547	0.188	0.01
ApoB/ApoA1	0.6 (0.22)	0.57 (0.19)	0.56 (0.14)	0.185	0.749	0.179
Homocysteine (mg/L)	3.82 (8.1)	2.4 (1.6)	2.01 (0.6)	0.063	0.06	0.137
Carotid ultrasound						
Atheromatous plaque, *n* (%)	51 (34.4)	33 (20.9)	3 (7.5)	0.002	0.102	0.006
CIMT	0.64 (0.2)	0.55 (0.12)	0.57 (0.13)	0.015	0.428	<0.001
Treatment						
bDMARD, *n* (%)	76 (51.3)	3 (1.9)	-	-	-	<0.001
csDMARD, *n* (%)	126 (85.1)	20 (12.6)	-	-	-	<0.001
Methotrexate, *n* (%)	92 (62.2)	3 (1.9)	-	-	-	<0.001
Sulfasalazine, *n* (%)	0	19 (12.1)	-	-	-	<0.001
Statins, *n* (%)	35 (23.6)	17 (10.8)	0	<0.001	0.029	0.002
Corticosteroids, *n* (%)	83 (56.1)	3 (1.9)	-	-	-	<0.001
Corticosteroid dose at cut-off	3.7 (4.2)	0.09 (0.7)	-	-	-	<0.001

Abbreviations: SD: Standard Deviation; BMI, body mass index; CV, cardiovascular; ACPA, anticitrullinated peptide antibody; DAS28, 28-joint Disease Activity Score; ESR, erythrocyte sedimentation rate; CRP, C-reactive protein; SDAI, Simple Disease Activity Index; CDAI, Clinical Disease Activity Index; SpA, spondyloarthritis; RA: Rheumatoid Arthritis; ASDAS, Ankylosing Spondylitis Disease Activity Score; BASDAI, Bath Ankylosing Spondylitis Disease Activity Index; BASFI, Bath Ankylosing Spondylitis Functional Index; VAS, visual analog scale; CIMT, carotid intima media thickness; NSAID, nonsteroidal anti-inflammatory drug; bDMARD, biological disease-modifying antirheumatic drugs; csDMARD, conventional synthetic disease-modifying antirheumatic drug; LDL, low-density lipoprotein; HDL, high-density lipoprotein.

**Table 2:** Multivariate linear regression model with patient sample.

Dependent Variable	Predictor	β Coefficient (95% CI)	*p*-Value
CIMT	Diagnosis (RA)	0.045 (0.0004 to 0.09)	0.048
	Male sex	0.069 (0.04 to 0.101)	<0.001
	Age (years)	0.005 (0.003 to 0.006)	<0.001
	Disease course	0.000 (−0.002 to 0.001)	0.444
	Statins	−0.018 (−0.007 to 0.034)	0.484
	Corticosteroids	−0.004 (−0.04 to 0.035)	0.807
	Smoking	0.031 (0.001 to 0.061)	0.037
	Arterial hypertension Dyslipidemia	0.068 (0.027 to 0.108) 0.036 (−0.009 to 0.082)	0.001 0.122

R^2^ = 0.405. CIMT: carotid intima media thickness; RA: Rheumatoid Arthritis.

**Table 3:** Multivariate linear regression model for patients classed as remission–low activity (*n* = 153).

Dependent Variable	Predictor	β Coefficient (95% CI)	*p*-Value
CIMT	Diagnosis (RA)	0.020 (−0.039 to 0.080)	0.500
	Male sex	0.077 (0.035 to 0.120)	<0.001
	Age (years)	0.005 (0.003 to 0.008)	<0.001
	Disease course	−0.002 (−0.004 to 0.000)	0.037
	Statins	−0.081 (−0.146 to 0.017)	0.013
	Corticosteroids	0.004 (−0.043 to 0.05)	0.839
	Smoking	0.011 (−0.028 to 0.052)	0.572
	Arterial hypertension Dyslipidemia	0.093 (0.041 to 0.146) 0.048 (−0.009 to 0.105)	<0.001 0.099

R^2^ = 0.504. CIMT: carotid intima media thickness; RA: Rheumatoid Arthritis.

**Table 4:** Multivariate linear regression model for patients classed as moderate–high activity (*n* = 138).

Dependent Variable	Predictor	β Coefficient (95% CI)	*p*-Value
CIMT	Diagnosis (RA)	0.112 (0.013–0.212)	0.026
	Male sex	0.058 (0.004–0.112)	0.034
	Age (years)	0.004 (0.001–0.006)	0.001
	Disease course	0.0004 (−0.002 to 0.002)	0.719
	Statins	0.029 (−0.070 to 0.129)	0.559
	Corticosteroids	−0.082 (−0.168 to 0.004)	0.061
	Smoking	0.045 (−0.006 to 0.097)	0.087
	Arterial hypertension Dyslipidemia	0.046 (−0.025 to 0.118) 0.041 (−0.044–0.125)	0.205 0.343

R^2^ = 0.374. CIMT: carotid intima media thickness; RA: Rheumatoid Arthritis.

## Data Availability

Data are available upon a reasonable request.

## Supplementary Materials

The following supporting information can be downloaded at: https://www.mdpi.com/article/10.3390/jcm11030662/s1, Table S1: Logistic regression models (DV: atheromatous plaque) according to inflammatory activity.
