# Supplementary material for: Subclinical Atherosclerosis Measure by Carotid Ultrasound and Inflammatory Activity in Patients with Rheumatoid Arthritis and Spondylarthritis
Source: J Clin Med. 2022 Jan 27;11(3):662. doi: 10.3390/jcm11030662 (PMC8836873; doi:10.3390/jcm11030662)
Supplement: Supplementary file 1 [file jcm-11-00662-s001.zip › jcm-1546482-supplementary.pdf]

Journal of
Clinical Medicine

MDPI

Table S1. Logistic regression models (DV: atheromatous plaque) according to inflammatory activity.

	**Atheromatous plaque**							
	**Remission-low activity (n=153)**				**Moderate-high activity (n=148)**			
	**Crude OR (95% CI)**	***p-value***	**Adjusted OR (95% CI)**	***p-value***	Crude OR (95% CI)	*p-value*	Adjusted OR (95% CI)	*p-value*
Diagnosis (RA)	2.57 (1.18-5.98)	**0.021**	2.24 (0.36-14.07)	0.381	1.77 (0.82-3.78)	0.141	1.69 (0.21-14.01)	0.619
Male sex	1.14 (1.09-1.19)	**<0.001**	1.15 (1.07-1.25)	**<0.001**	1.16 (1.1-1.24)	**<0.001**	1.19 (1.09-1.33)	**<0.001**
Age (years)	2.20 (1.07-4.61)	**0.034**	12.4 (3.37-57.6)	**<0.001**	1.09 (0.52-2.31)	0.808	0.75 (0.22-2.45)	0.643
Disease course	1 (0.97-1.04)	0.651	1.02 (0.95-1.09)	0.495	1.06 (1.03-1.09)	**<0.001**	1.05 (0.99-1.11)	0.064
Statins	3.07 (1.29-7.3)	**0.010**	1.48 (0.42-5.17)	0.529	3.38 (1.29-8.95)	**0.012**	3.58 (0.77-19.5)	0.116
Corticosteroids	2.29 (1.07-4.87)	**0.030**	1.78 (0.40-8.25)	0.443	0.83 (0.35-1.9)	0.679	0.20 (0.03-1.14)	0.076
Smoking	4.49 (2.09-9.85)	**<0.001**	6.49 (2.04-23.3)	**0.002**	1.16 (0.54-2.51)	0.689	6.63 (1.87-28.5)	**0.006**
Arterial hypertension	4.10 (1.85-9.24)	**<0.001**	1.22 (0.32-4.72)	0.766	4.81 (2.12-11.16)	**<0.001**	0.97 (0.25-3.66)	0.970

Abbreviations: RA, rheumatoid arthritis; OR, odds ratio.
